# Concordance between administrative claims and registry data for identifying metastasis to the bone: an exploratory analysis in prostate cancer

**DOI:** 10.1186/1471-2288-14-1

**Published:** 2014-01-02

**Authors:** Eberechukwu Onukwugha, Candice Yong, Arif Hussain, Brian Seal, C Daniel Mullins

**Affiliations:** 1 Pharmaceutical Health Services Research Department, University of Maryland School of Pharmacy, 220 Arch Street, Baltimore, MD 21201, USA; 2 Department of Medicine, University of Maryland School of Medicine, 655 West Baltimore Street, Baltimore, MD 21201, USA; 3 Baltimore Veterans Affairs Medical Center, 655 West Baltimore Street, Baltimore, MD 21201, USA; 4 Bayer Healthcare Pharmaceuticals, 6 West Belt, Wayne, NJ 07470-6806, USA

**Keywords:** Metastasis, Bone, SEER, Claims, Concordance

## Abstract

**Background:**

To assess concordance between Medicare claims and Surveillance, Epidemiology, and End Results (SEER) reports of incident BM among prostate cancer (PCa) patients. The prevalence and consequences of bone metastases (BM) have been examined across tumor sites using healthcare claims data however the reliability of these claims-based BM measures has not been investigated.

**Methods:**

This retrospective cohort study utilized linked registry and claims (SEER-Medicare) data on men diagnosed with incident stage IV M1 PCa between 2005 and 2007. The SEER-based measure of incident BM was cross-tabulated with three separate Medicare claims approaches to assess concordance. Sensitivity, specificity and positive predictive value (PPV) were calculated to assess the concordance between registry- and claims-based measures.

**Results:**

Based on 2,708 PCa patients in SEER-Medicare, there is low to moderate concordance between the SEER- and claims-based measures of incident BM. Across the three approaches, sensitivity ranged from 0.48 (0.456 – 0.504) to 0.598 (0.574 - 0.621), specificity ranged from 0.538 (0.507 - 0.569) to 0.620 (0.590 - 0.650) and PPV ranged from 0.679 (0.651 - 0.705) to 0.690 (0.665 - 0.715). A comparison of utilization patterns between SEER-based and claims-based measures suggested avenues for improving sensitivity.

**Conclusion:**

Claims-based measures using BM ICD 9 coding may be insufficient to identify patients with incident BM diagnosis and should be validated against chart data to maximize their potential for population-based analyses.

## Background

Among men diagnosed with prostate cancer (PCa), seventy to eighty percent of those with metastatic disease have involvement of the bone [1-4] with significant implications for pain, morbidity and mortality [2,5-8]. Increasingly, researchers are using claims-based measures of bone metastasis (BM) to examine incidence, associated costs, and survival [4,6,7,9,10]. These real world data, including the billing codes such as the International Classification of Diseases, 9^th^ Revision Clinical Modification (ICD-9-CM) codes, reflect clinical practice but do not provide a consistent means of verifying the accuracy of clinical diagnoses. Using the Surveillance, Epidemiology and End Results (SEER) registry and linked Medicare claims available from the National Cancer Institute, we undertook the present study in an effort to better understand the concordance between registry-based data on BM and claims-based measures of BM, using men diagnosed with incident metastatic PCa as a model. To our knowledge, this is the first study to investigate the agreement between claims-based and registry-based sources of BM.

Evidence regarding the validity of using claims data to identify cancer stage, progression, and metastasis is not favorable [11-13]. Moreover, the validity of using claims data to identify patients with BM may differ depending on the approach used. Previous studies have identified patients with BM based on the presence of a diagnosis of “secondary malignant neoplasm of bone and bone marrow” (ICD-9-CM 198.5) in claims data. These claims-based approaches differ in terms of the incorporation of the ICD-9-CM codes, for example, whether the codes should be present alone or with other procedure codes used to diagnose or treat BM. Several studies have defined BM patients as persons with two or more encounters including 198.5 anytime on or after the date of the first claim with a diagnosis of cancer [4,9,10]. Other studies have defined BM patients as persons with at least one inpatient claim with the 198.5 code, at least one outpatient claim with the 198.5 code paired with a code for procedures used to diagnose or treat BM, or at least one outpatient physician evaluation and management claim with the 198.5 code [6,7].

Prior studies have reported BM prevalence using SEER cancer registries data linked with Medicare enrolment and claims files. The SEER data has traditionally provided AJCC metastasis information to confirm the incident staging of M1 (distant metastasis) or M0 (no distant metastasis). Starting in 2004, SEER adopted the Collaborative Stage (CS) system and SEER registries started to provide detail regarding the sub-stages of M1 disease: M1a (non-regional lymph nodes), M1b (bone), and M1c (other site, with or without bone disease). This SEER variable has not been validated and is generally not considered a gold standard for identification of the site of metastatic disease. As researchers consider its use in population studies involving SEER-Medicare data, information regarding the agreement between the M1b measure and claims-based data will be important to consider. The availability of registry-based information regarding incident BM diagnosis from SEER provides the opportunity to investigate the agreement between claims-based and registry-based measures of BM.

The objective of this study was to determine the concordance between the SEER registry measure of an incident BM diagnosis and the claims-based measures of BM-related health services utilization around the time of diagnosis. A secondary objective was to identify claims-based measures that could enrich claims-based BM approaches. These objectives are intended to support consistency in the use of claims-based BM approaches and support a more transparent and reliable approach to the development of claims-based approaches for studying cancer treatments and outcomes.

## Methods

### Data

This retrospective analysis of linked cancer registry and Medicare claims data included men at least 66 years of age diagnosed with incident PCa between 2005 and 2007 as listed in the SEER cancer registry. Cases were limited to those diagnosed with stage IV metastatic (M1) disease as identified by the American Joint Committee on Cancer Tumor-Node-Metastasis (AJCC-TNM) stage, 6^th^ edition [14]. Claims data from 2004 to 2009 were extracted from linked Medicare claims files. The requirement for continuous enrollment in Medicare Parts A and B during the 12 months prior to and including the month of diagnosis constituted an additional inclusion criterion. Exclusion criteria were: 1) health maintenance organization (HMO) enrollment during the 12 months prior to and including the month of diagnosis since HMO claims can be unreliable due to missing data; 2) history of other cancers within 5 years prior to PCa diagnosis. Patients were censored if they enrolled in an HMO or lost Part A and/or B enrollment at any time following the diagnosis date, or if the end of the study period (December, 2009) was reached. This study was approved by the University of Maryland Baltimore Institutional Review Board (#HP-00049426).

### Variables

#### Measures of bone metastasis diagnosis or associated health utilization

**Figure 1:**
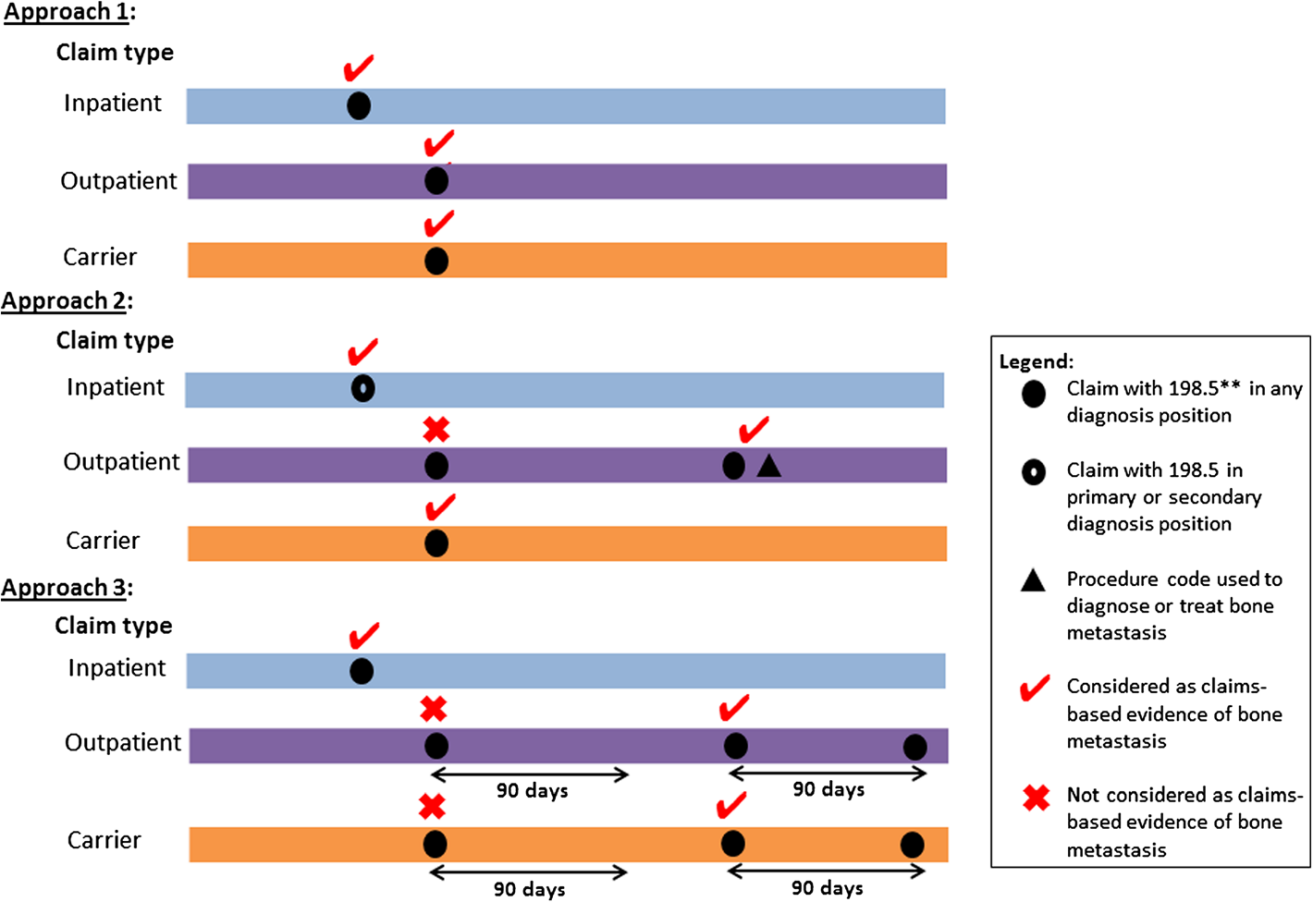
Identification of patients with bone metastasis* using Medicare inpatient, outpatient, and carrier claims. *Patient identified to have bone metastasis if patient has claims-based evidence of bone metastasis from inpatient or outpatient or carrier claims. **ICD-9 diagnosis code of 198.5 represents ‘secondary malignant neoplasm of bone and bone marrow’.

Patients were identified as having a SEER-based measure of BM if the AJCC metastatic component in the Collaborative Stage (CS) coding system indicated ‘M1b’ status, i.e. metastasis to bone at diagnosis. In defining the study cohort, we excluded the first year (i.e. 2004) in which the M1b measure became available in order to avoid possible coding problems that could have arisen as cancer registries gained familiarity with furnishing the M1b code. We investigated differences between three claims-based approaches to identify patients with BM-related claims (see Figure 1). We created a ‘generous’ approach (Approach 1), adopted an approach that is similar to the approach used in previous studies [6,7] (Approach 2), and created a more restrictive approach (Approach 3) as follows:

Approach 1

At least one inpatient, outpatient, or carrier claim with an ICD-9 diagnosis code of 198.5 (‘secondary malignant neoplasm of bone and bone marrow’) in any diagnosis field.

Approach 2

At least one inpatient claim with an ICD-9 diagnosis code of 198.5 as the primary or secondary discharge diagnosis; *OR* at least one outpatient claim with a diagnosis code of 198.5 paired with a code for procedures used to diagnose or treat BM such as bone scan, bone biopsy, and/or use of intravenous bisphosphonate; *OR* at least one outpatient physician claim with a diagnosis code of 198.5.

Approach 3

At least one inpatient claim with an ICD-9 diagnosis code of 198.5 in any diagnosis field; *OR* at least two outpatient claims within a 90-day window with a diagnosis code of 198.5.

**Figure 2:**
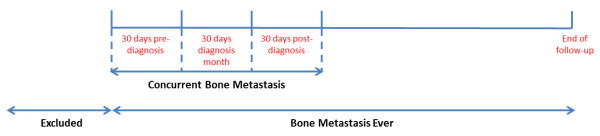
Identification of concurrent bone metastasis and bone metastasis ever using claims data.

For each of the three approaches, patients were classified as having concurrent BM-related claims if claims submitted in the month before, during, or after the month of PCa diagnosis satisfied the condition stipulated by the approach. The exact date of diagnosis is not available from the SEER data and Medicare claims relevant to an event occurring in a particular month can appear in the month prior to and following the month in which the event occurred [15]. Figure 2 provides a graphical representation of ‘concurrent BM’-related claims, i.e. BM-related claims that were considered to be concurrent with the PCa diagnosis. The 3-month (90-day) window has been used in previous studies to define concurrent BM [6].

#### Demographics and health care utilization measures

Patient-level demographic and clinical variables obtained from the SEER files include age, race, marital status, urban residence, prostate specific antigen (PSA) level and tumor differentiation at diagnosis. We assessed comorbid illness using the Charlson Comorbidity Index (CCI) [16] and the National Cancer Institute (NCI) Combined Index [17] using claims from the 12-month period before the month of diagnosis. Treatment receipt, use of health services such as bone biopsy, and bone or joint imaging, PSA tests, and cancer specialist visits were identified from MEDPAR and Part B claims.

### Statistical analysis

Cross-tabulations of the claims-based BM approaches and the SEER-based measure of BM were used to compare concordance. We calculated sensitivity, specificity, and positive predictive value (PPV) for each approach compared to the M1b measure from SEER. Sensitivity for each claims-based approach was calculated as the proportion of patients with a SEER-based BM diagnosis who were identified to have BM-related utilization in Medicare claims. Specificity for each claims-based approach was calculated as the proportion of patients without a SEER-based BM diagnosis who also did not have BM-related utilization in Medicare claims. Positive predictive value was calculated as the proportion of patients with claims-based BM-related utilization who had incident BM diagnosis based on registry data.

In order to investigate the possibilities for improving sensitivity, we selected the measure with the lowest sensitivity for use in subsequent analyses. The chi-square test identified statistically significant differences in health services utilization between patients grouped with respect to: (1) presence or absence of concurrent BM-related health services utilization according to the claims-based approach; and (2) presence or absence of BM at diagnosis according to the SEER-based measure of BM.

To identify additional measures that could enhance the sensitivity of claims-based approaches, the sample with SEER-based evidence of BM was stratified by the presence or absence of concurrent BM according to claims-based Approach 3. Among this sample of patients with a diagnosis of BM based on registry data, the objective was to identify health resource utilization categories that are commonly reported among patients without BM-related claims. Utilization categories meeting these criteria can be used to improve the sensitivity of definitions created to identify men with an incident diagnosis of BM and/or with a diagnosis of BM outside the diagnosis window using health care claims data. We conducted sensitivity analysis focused on improving the sensitivity of Approach 3.

## Results

### Descriptive results

After applying study inclusion and exclusion criteria, the final study sample included 2,708 men diagnosed with incident stage IV metastatic PCa. Descriptive statistics for the full sample are presented in Table 1. The concordance between the two measures was captured using sensitivity, specificity, and PPV. The sensitivity, specificity, and PPV of the three claims-based approaches compared to the SEER-based measure of BM are presented in Table 2. The receipt of radiation (any type), external beam radiation therapy, radiopharmaceutical therapy, and intravenous bisphosphonate therapy at any time following diagnosis was higher among individuals with BM according to either SEER-based or claims-based measures. In terms of diagnostic tests, the receipt of bone or joint imaging at any time following diagnosis was higher among individuals with BM according to either SEER-based or claims-based measures. The SEER-based and claims-based measures were not consistent in terms of the relationship between physician visits (i.e., medical oncologists, radiation oncologists) and BM.

**Table 1:** Descriptive statistics for men diagnosed with metastatic prostate cancer between 2005 and 2007 (N = 2,708)

	** N**	** %**
Race/Ethnicity		
White non-Hispanic	2096	77.4
African American non-Hispanic	326	12.0
Hispanic	159	5.9
Other	127	4.7
Age		
66-69	375	13.9
70-74	499	18.4
75-79	555	20.5
80-84	645	23.8
85+	634	23.4
Married	1583	62.3
Urban location	2396	88.5
High PSA at baseline	2298	84.9
Poorly differentiated tumor	1638	60.5
Charlson Comorbidity Index		
Zero	1453	53.7
One	547	20.2
Two or higher	470	17.4
Missing	238	8.8
National Cancer Institute (NCI) Combined Index		
Zero	1459	54.2
0-2	943	35.0
> = 2	53	2.0
Missing	238	8.8
Bone metastasis		
*Bone metastasis diagnosis*		
SEER registry	1694	62.6
*Bone metastasis – related utilization*		
Claims – BM Concurrent, Approach 1	1481	54.7
Claims – BM Concurrent, Approach 2	1363	50.3
Claims – BM Concurrent, Approach 3	1198	44.2

**Table 2:** Sensitivity, specificity, and positive predictive value (PPV) of three claims-based measures defined concurrently with diagnosis of prostate cancer

	**Claims-based measure of BM-related utilization concurrent with diagnosis**		
	**Approach 1**	**Approach 2**	**Approach 3**
Sensitivity (95% CI*)	0.598 (0.574 - 0.621)	0.555 (0.531 - 0.579)	0.480 (0.456 - 0.504)
Specificity (95% CI*)	0.538 (0.507 - 0.569)	0.584 (0.553 - 0.614)	0.620 (0.590 - 0.650)
PPV (95% CI*)	0.684 (0.660 - 0.708)	0.690 (0.665 - 0.715)	0.679 (0.651 - 0.705)

*95% confidence intervals were calculated using the Clinical research calculator from *VassarStats*[18] based on: Newcombe, Robert G. “Two-Sided Confidence Intervals for the Single Proportion: Comparison of Seven Methods”, Statistics in Medicine, 17, 857–872 (1998).

### Subgroup comparisons based on health care utilization in full sample

Approach 3 was considered to be the best approach amongst the three options because, relative to the other two approaches, it relaxed the criteria based on inpatient claims (the coding of which is generally considered reliable) and at the same time tightened the criteria based on outpatient claims (the coding of which may be problematic for identifying clinical conditions). Approach 3 had the highest specificity, and thus performed best at excluding individuals who were false positives. On the other hand, it had the lowest sensitivity, i.e. a higher number of false negatives. Subsequent analyses sought to identify measures that could be used to supplement Approach 3, with the goal of reducing the number of false negatives.

Table 3 shows the proportion of patients with post-diagnosis health services utilization in terms of diagnostic testing/surveillance procedures and physician visits, stratified by presence of claims-based concurrent BM-related utilization and presence of SEER-based incident BM diagnosis. Proportions were reported as column percentages.

**Table 3:** Proportion of patients with post-diagnosis health services utilization, stratified by alternative bone metastasis measures

			**Claims Approach 3**					**SEER measure**				
	**Full M1 sample**		**Concurrent BM**		**No concurrent BM**			**BM at diagnosis**		**No BM at diagnosis**		
	**N = 2,708**		**N = 1,198**		**N = 1,510**			**N = 1,694**		**N = 1,014**		
			**(44.2%)**		**(55.8%)**			**(62.6%)**		**(37.4%)**		
	** N**	** Col%**	** N**	** Col%**	** N**	** Col%**	**P-value**	** N**	** Col%**	** N**	** Col%**	**P-value**
**Post-diagnosis resource utilization (i.e., till end of follow-up)**												
Bone mineral density (BMD)	109	4.0	40	3.3	69	4.6	0.11	71	4.2	38	3.8	0.57
Test												
PSA test	2,148	79.3	906	75.6	1,242	82.3	<0.01	1,389	82.0	759	74.9	<0.01
Oncologist visit	1,681	62.1	857	71.5	824	54.6	<0.01	1,041	61.5	640	63.1	0.39
Nuclear medicine specialist	NR	NR	NR	+	NR	-	0.07	NR	>0	NR	0.0	0.01
Visit												
Radiation oncologist visit	1,066	39.4	528	44.1	538	35.6	<0.01	687	40.6	379	37.4	0.10
Bone biopsy	79	2.9	62	5.2	17	1.1	<0.01	47	2.8	32	3.2	0.57
Bone or joint imaging	2,200	81.2	1,006	84.0	1,194	79.1	<0.01	1,406	83.0	794	78.3	<0.01
**Treatment receipt (Part B)**												
Radiation	889	32.8	445	37.2	444	29.4	<0.01	581	34.3	308	30.4	0.04
External Beam radiation	839	31.0	425	35.5	414	27.4	<0.01	553	32.6	286	28.2	0.02
Therapy												
Radiopharmaceutical therapy	91	3.4	60	5.0	31	2.1	<0.01	72	4.3	19	1.9	<0.01
Bisphosphonates IV	862	31.8	494	41.2	368	24.4	<0.01	593	35.0	269	26.5	<0.01
Erythropoietin	535	19.8	274	22.9	261	17.3	<0.01	352	20.8	183	18.1	0.08
Opioids (moderate-severe)	624	23.0	248	20.7	376	24.9	0.01	379	22.4	245	24.2	0.28
			**Claims Approach 3**					**SEER measure**				
	**Full M1 sample**		**Concurrent BM**		**No concurrent BM**			**BM at diagnosis**		**No BM at diagnosis**		
	**N = 2,708**		**N = 1,198**		**N = 1,510**			**N = 1,694**		**N = 1,014**		
			**(44.2%)**		**(55.8%)**			**(62.6%)**		**(37.4%)**		
	** N**	** Col%**	** N**	** Col%**	** N**	** Col%**	**P-value**	** N**	** Col%**	** N**	** Col%**	**P-value**
**Resource utilization during the 90-day diagnosis period**												
PSA test	1,629	60.2	698	58.3	931	61.7	0.07	1,046	61.8	583	57.5	0.03
Bone biopsy	65	2.4	NR	+	NR	-	<0.01	42	2.5	23	2.3	0.73
Bone or joint imaging	2,009	74.2	999	83.4	1,010	66.9	<0.01	1,317	77.7	692	68.2	<0.01
	**Mean**	**S.D.**	**Mean**	**S.D.**	**Mean**	**S.D.**	**p-value**	**Mean**	**S.D.**	**Mean**	**S.D.**	**p-value**
**Post-diagnosis resource utilization (i.e., till end of follow-up)**												
Number of BMD tests	0.04	0.21	0.03	0.19	0.05	0.22	0.09	0.04	0.22	0.04	0.19	0.39
Number of PSA tests	8.16	10.01	8.26	10.77	8.08	9.38	0.63	8.81	10.41	7.07	9.21	<0.01
Number of PSA tests among patients with PSA tests	10.29	10.23	10.93	11.15	9.82	9.47	0.02	10.75	10.56	9.44	9.54	<0.01
Number of bone biopsies	0.03	0.18	0.05	0.24	0.01	0.11	<0.01	0.03	0.18	0.03	0.19	0.53
Number of bone or joint imaging	1.65	1.58	1.67	1.56	1.64	1.61	0.60	1.73	1.61	1.52	1.53	<0.01
**Resource utilization during the 90-day diagnosis period**												
Number of PSA tests	0.89	0.92	0.92	1.01	0.86	0.84	0.07	0.92	0.94	0.83	0.89	0.01
Number of bone biopsies	0.02	0.16	0.05	0.23	0.003	0.06	<0.01	0.03	0.17	0.02	0.15	0.60
Number of bone or joint imaging	0.76	0.47	0.86	0.42	0.68	0.49	<0.01	0.79	0.44	0.70	0.49	<0.01

*NR*, Not reported due to small sample size, per data use agreement; ‘+’ means that the column% is greater than the percentage for the full sample while ‘-‘ means that the column% is smaller than the percentage for the full sample.

Examining percentages and how they differ across groups defined using the claims-based approach and the SEER-based measure facilitates the identification of measures that could be used to reduce the number of false negatives identified by the claims-based approach. The relevant measures would be positively associated with a BM diagnosis and negatively associated with claims-based evidence of BM-related utilization. Utilization of PSA tests and the intensity of use of PSA tests could be useful in this regard. The proportion of patients with a claim for a PSA test and the mean number of PSA claims per person were each statistically significantly higher among patients with SEER-based BM diagnosis compared to patients without SEER-based BM diagnosis when considering utilization at any time. In contrast, the proportion of patients with any PSA test at any time during the follow-up period was statistically significantly lower among patients with claims-based evidence of concurrent BM-related utilization compared to patients without claims-based evidence of concurrent BM-related utilization. Consideration of utilization during the diagnosis period, rather than at any time, could be particularly useful when the focus is on identifying individuals with incident BM. Results for tests or procedures occurring within the 90-day diagnosis period are provided in the last section of Table 3.

### Subgroup comparisons based on health care utilization among M1b patients

Differences between patients grouped according to concurrent claims-based BM-related utilization were examined among patients with an incident BM diagnosis. Utilization that is positively correlated with the M1b measure (Table 3) and negatively correlated with the concurrent claims-based BM-related utilization measure could be used to supplement Approach 3 so as to reduce false negatives. The likelihood and frequency of bone or joint imaging during the diagnosis period was higher among individuals with BM according to SEER (Table 3). Among the 1,694 patients, the likelihood and frequency of bone or joint imaging was higher during the diagnosis period and similar during the follow-up period when comparing individuals with and without BM according to Approach 3 (Table 4). The likelihood and frequency of PSA tests during the diagnosis period was higher when comparing individuals with and without BM according to SEER (Table 3). Among the 1,694 patients and during either the diagnosis or follow-up periods, the likelihood of a PSA test was lower and the frequency of PSA testing was not statistically significantly different when considering Approach 3 (Table 4).

**Table 4:** Health services utilization among patients with SEER-based evidence of BM (M1b), stratified by presence or absence of concurrent BM according to claims-based Algorithm 3

	**M1b Sample**		**Concurrent BM claims algorithm 3**		**No concurrent BM claims algorithm 3**		**p-value**
	**N = 1,694**		**N = 813**		**N = 881**		
			**(48.0%)**		**(52.0%)**		
	** N**	**Col%**	** N**	**Col%**	** N**	**Col%**	
**Post-diagnosis resource utilization (i.e., till end of follow-up)**							
Bone mineral density (BMD) test	71	4.2	28	3.5	43	4.9	0.14
PSA test	1,389	82.0	640	78.7	749	85.0	<0.01
Oncologist visit	1,041	61.5	567	69.7	474	53.8	<0.01
Nuclear medicine specialist visit	NR	NR	NR	+	NR	-	0.10
Radiation oncologist visit	687	40.6	369	45.4	318	36.1	<0.01
Bone biopsy	47	2.8	NR	+	NR	-	<0.01
Bone or joint imaging	1,406	83.0	684	84.1	722	82.0	0.23
**Treatment receipt (Part B)**							
Radiation	581	34.3	315	38.8	266	30.2	<0.01
External beam radiation therapy	553	32.6	302	37.2	251	28.5	<0.01
Radiopharmaceutical therapy	72	4.3	45	5.5	27	3.1	0.01
Bisphosphonates IV	593	35.0	351	43.2	242	27.5	<0.01
Erythropoietin	352	20.8	192	23.6	160	18.2	0.01
Opioids (moderate-severe)	379	22.4	167	20.5	212	24.1	0.08
**Resource utilization during the 90-day diagnosis period**							
PSA test	1,046	61.8	481	59.2	565	64.1	0.04
Bone biopsy	42	2.5	NR	+	NR	-	<0.01
Bone or joint imaging	1317	77.7	690	84.9	627	71.2	<0.01
	**Mean**	**S.D.**	**Mean**	**S.D.**	**Mean**	**S.D.**	**p-value**
**Post-diagnosis resource utilization (i.e., till end of follow-up)**							
Number of BMD tests	0.04	0.22	0.04	0.19	0.05	0.24	0.11
Number of PSA tests	8.81	10.41	8.80	10.90	8.83	9.95	0.95
Number of bone biopsies	0.03	0.18	0.05	0.24	0.01	0.09	<0.01
Number of bone or joint imaging	1.73	1.61	1.70	1.56	1.75	1.66	0.51
**Resource utilization during the 90-day diagnosis period**							
Number of PSA tests	0.92	0.94	0.95	1.04	0.89	0.83	0.18
Number of bone biopsies	0.03	0.17	0.05	0.24	0.003	0.06	<0.01
Number of bone or joint imaging	0.79	0.44	0.87	0.41	0.72	0.47	<0.01

*NR*, Not reported due to small sample size, per data use agreement; ‘+’ means that the column% is greater than the percentage for the full sample while ‘-‘means that the column% is smaller than the percentage for the full sample.

With the focus on improving the sensitivity of Approach 3 based on results in Table 3, we expanded the definition of Approach 3 to include situations where there were two outpatient claims during the diagnosis period for a PSA test or a bone/joint imaging test. The tests had to occur within 90 days of each other. Following this exercise, sensitivity of the expanded Approach 3 was improved: 0.581 (0.558 – 0.605) compared to 0.48 (0.456 – 0.504) for the original Approach 3. The specificity of the updated Approach 3 was reduced: 0.558 (0.527 – 0.589) compared to 0.62 (0.59 – 0.65) for the original Approach 3. Changes to the algorithm focused on specificity also can be identified and implemented.

## Discussion

Evaluation of the incidence and impact of BM among cancer patients requires reliable estimation of BM. We found that there is low to moderate concordance between the SEER-based and claims-based measures of bone metastasis (BM) in a sample of men diagnosed with incident advanced disease. We conducted the analysis using data on men diagnosed with incident advanced PCa although the investigation would be relevant to any study using healthcare claims data to investigate the occurrence of BM among individuals diagnosed with advanced stage cancer. We found that inconsistency in terms of the absence of incident BM diagnosis according to the registry data and the presence of a baseline BM diagnosis according to the claims data occurred when a generous (i.e. catch-all) claims-based measure was employed. The greatest potential for missing patients with an incident diagnosis of BM according to the registry data occurred when employing a restrictive claims-based measure. Our study leveraged the availability of SEER-based and claims-based information regarding the same clinical event, i.e. the diagnosis of BM in the registry data and health care utilization that is ostensibly related to either diagnosis or treatment of BM in the Medicare claims data.

Medicare claims constitute a rich source of information for investigating treatment utilization and management over time for patients with continuous Medicare coverage. When linked with cancer registry data, the claims data provide important information regarding treatment and management following the cancer diagnosis. The potential benefits of claims data have to be considered in the context of some of the limitations, including the limited ability to confirm the presence of clinically diagnosed conditions. In this paper, we focused on the diagnosis of BM among elderly men with PCa given the implications of a BM diagnosis on patient quality of life [19], prognosis [6,19,20], and treatment costs [4]. When the BM diagnosis occurs concurrently with the PCa diagnosis, post-diagnosis cancer care shifts dramatically to an increased focus on bone health, pain management, and quality of life. The BM diagnosis can also occur after the initial diagnosis of PCa, with often severe implications for the patient’s health. Thus, it would be important to be able to reliably identify the population of patients with BM using generalizable data such as SEER-Medicare.

The SEER-Medicare cohort included men diagnosed with PCa from 2005 to 2007, providing the opportunity to investigate the concordance between the data regarding an incident BM diagnosis supplied by the cancer registries and information from the claims data regarding BM-related health care utilization around the diagnosis period. We excluded the 2004 cohort since that was the first year that information regarding a BM diagnosis was available from the SEER registry. Approach 1 was created based on the rationale that coding for BM on a health care claim would occur only when the patient had a diagnosis of BM. Approach 3 was created based on the rationale that: 1) in the inpatient setting, a hospitalized individual with a BM diagnosis may not necessarily be hospitalized as a result of their specific BM diagnosis, and therefore the diagnosis code of BM could appear in any position within the diagnosis fields; and 2) in the outpatient setting, a claim for a service that could be used to diagnose (or rule out) BM may be more useful if at least two claims were required to be more certain that BM was present.

None of the approaches in Table 2 was uniformly superior and given the focus on identifying the concordance between available measures of bone metastasis, the next step with respect to the development of reliable claims-based measures would be to provide guidance on the avenues for improving their reliability. Based on information in Table 3 regarding utilization during the diagnosis period, the frequency of PSA testing and the frequency of bone/joint imaging was higher among individuals with incident BM diagnosis according to the SEER registry data compared to individuals who were not identified in SEER as having BM. The higher testing frequency may reflect more intense follow-up schedules involving specific tests after a diagnosis of BM compared to patients who do not have a BM diagnosis. The results from this exercise indicated that it is possible to improve the sensitivity of claims-based measures. Results also suggest that the informative measures that emerge when analyzing data within a retrospective study design will not be limited to ‘predictive’ variables and that researchers may also draw inference from utilization patterns that occur following the BM diagnosis.

There are some limitations that also need to be considered. There has been no validation of the SEER registry M1b measure and so its measurement properties are not fully understood. We excluded registry data on M1b for the 2004 cohort year however some inaccuracies in M1b coding could still be present in subsequent years. Reliance on ICD 9 diagnosis coding for BM could be problematic when examining outpatient claims for diagnostic tests and procedures. Claims associated with services intended to rule out BM should not include the 198.5 code on the claim since the BM diagnosis is not established. A two-step approach for including diagnostic tests/procedures based on diagnosis codes may be: 1) examine all claims regardless of whether or not they have a BM ICD 9 code; 2) include only those diagnostic claims that are followed (e.g. within 90 days) by a claim of 198.5.

The comparison undertaken in this study is instructive for two important reasons: 1) as noted in the introductory text, claims-based measures are already in use by researchers to investigate the clinical and economic burden of BM across various disease sites and will remain a source for population-based, real-world evidence regarding prevalence, utilization, and outcomes associated with metastasis to the bone; 2) the linked cancer registry data provide unique clinical, cancer-specific information and are generally considered to be more reliable than claims data for confirming clinical diagnoses (e.g., AJCC M1 staging information available in SEER compared with ICD 9 codes for distant metastasis). However, information regarding disease progression and health utilization (e.g., treatment, physician visits, hospice use) is not available in registry data thus claims data will remain the source of information on utilization among incident and prevalent BM cases across cancer sites including prostate cancer, lung cancer, and breast cancer. From a public health standpoint focused on improving health outcomes for men and women with advanced cancer, it will be important to develop validated measures of a BM diagnosis using claims-based data.

## Conclusion

We identified low to moderate concordance between the Medicare claims anchoring on codes used for diagnosing bone metastasis and the SEER registry data that is indicative of incident diagnosis of bone metastasis. Researchers utilizing the SEER or linked SEER datasets to investigate bone metastasis should exercise caution given the low agreement between the two sources of information regarding an incident diagnosis of bone metastasis. Until further research provides a validated claims-based approach to identifying BM, it is prudent to focus on individuals with metastatic disease and not seek to subset the population further based on metastasis to the bone. Claims-based approaches should be validated against chart data to maximize their potential for population-based analyses.

## Abbreviations

AJCC: American joint committee on cancer; BM: Bone metastasis; CCI: Charlson Comorbidity Index; CS: Collaborative stage; HMO: Health Maintenance Organization; ICD 9 CM: International classification of diseases, 9^th^ revision clinical modification; MEDPAR: Medicare provider analysis and review; NCI: National Cancer Institute; PCa: Prostate cancer; PPV: Positive predictive value; PSA: Prostate-specific antigen; SEER: Surveillance, epidemiology, and end results; TNM: Tumor, node, metastasis.

## Competing interests

EO: grant support from Bayer, Pfizer, sanofi-aventis, and Novartis; consulting income from Pfizer and Janssen/Johnson & Johnson.

CY: support from Bayer.

BS: stock holder and employee of Bayer.

AH: grant support from Bayer and Amgen.

CDM: grant support from Bayer and Pfizer; consulting income from Amgen, Bayer, BMS, Celgene, GSK. Janssen/Johnson & Johnson, Mitsubishi, Novartis, and Pfizer.

## Authors’ contributions

EO designed the study, contributed to the acquisition of data and coordination of the study, contributed to the interpretation of results, and drafting of the manuscript. CY performed the statistical analysis, contributed to interpretation of the data, and drafting of the manuscript. BS participated in the coordination of the study and contributed to revising the manuscript and reviewing for important intellectual content. CDM contributed to acquisition of the data, participated in the coordination of the study, and contributed to revising the manuscript for critical intellectual content. AH contributed to the design of the study and revision of the manuscript for critical intellectual content. All authors read and approved the final manuscript.

## Pre-publication history

The pre-publication history for this paper can be accessed here:

http://www.biomedcentral.com/1471-2288/14/1/prepub

## References

[B1] Barlev A Song X Ivanov B Setty V Chung K Payer costs for inpatient treatment of pathologic fracture, surgery to bone, and spinal cord compression among patients with multiple myeloma or bone metastasis secondary to prostate or breast cancer J Manag Care Pharm 2010 16 9 693 702 21067255 10.18553/jmcp.2010.16.9.693 PMC10437882

[B2] Bruera ED Sweeney C Bruera ED, Portenoy RK Bone pain Cancer Pain: Assessment and Management 2003 New York: Cambridge University Press 413 428

[B3] Groot MT Boeken Kruger CG Pelger RC Uyl-de Groot CA Costs of prostate cancer, metastatic to the bone, in the Netherlands Eur Urol 2003 43 3 226 232 10.1016/S0302-2838(03)00007-1 12600424

[B4] Lage MJ Barber BL Harrison DJ Jun S The cost of treating skeletal-related events in patients with prostate cancer Am J Manag Care 2008 14 5 317 322 18471035

[B5] Saad F Lipton A Cook R Chen YM Smith M Coleman R Pathologic fractures correlate with reduced survival in patients with malignant bone disease Cancer 2007 110 8 1860 1867 10.1002/cncr.22991 17763372

[B6] Sathiakumar N Delzell E Morrisey M Falkson C Yong M Chia V Blackburn J Arora T Kilgore M Mortality following bone metastasis and skeletal-related events among men with prostate cancer: a population-based analysis of US Medicare beneficiaries,1999-2006 Prostate Cancer Prostatic Dis 2011 14 177 183 10.1038/pcan.2011.7 21403668

[B7] Sathiakumar N Delzell E Morrisey MA Falkson C Yong M Chia V Blackburn J Arora T Brill I Kilgore ML Mortality following bone metastasis and skeletal-related events among women with breast cancer: a population-based analysis of U.S. Medicare beneficiaries, 1999–2006 Breast Cancer Res Treat 2011 131 1 231 238 21842243 10.1007/s10549-011-1721-x

[B8] Schulman KL Kohles J Economic burden of metastatic bone disease in the U.S Cancer 2007 109 11 2334 2342 10.1002/cncr.22678 17450591

[B9] Delea T McKiernan J Brandman J Edelsberg J Sung J Raut M Oster G Retrospective study of the effect of skeletal complications on total medical care costs in patients with bone metastases of breast cancer seen in typical clinical practice J Support Oncol 2006 4 7 341 347 16892696

[B10] Delea TE McKiernan J Brandman J Edelsberg J Sung J Raut M Oster G Impact of skeletal complications on total medical care costs among patients with bone metastases of lung cancer J Thorac Oncol 2006 1 6 571 576 10.1097/01243894-200607000-00012 17409919

[B11] Hassett MJ Ritzwoller DP Taback N Carroll N Cronin AM Ting GV Schrag D Warren JL Hornbrook MC Weeks JC Validating billing/encounter codes as indicators of lung, colorectal, breast, and prostate cancer recurrence using 2 large contemporary cohorts Med Care 2012 [Epub ahead of print] 10.1097/MLR.0b013e318277eb6f PMC3600389 23222531

[B12] Nordstrom BL Whyte JL Stolar M Mercaldi C Kallich JD Identification of metastatic cancer in claims data Pharmacoepidemiol Drug Saf 2012 21 Suppl 2 21 28 22552976 10.1002/pds.3247

[B13] Thomas SK Brooks SE Mullins CD Baquet CR Merchant S Use of ICD-9 coding as a proxy for stage of disease in lung cancer Pharmacoepidemiol Drug Saf 2002 11 8 709 713 10.1002/pds.759 12512248

[B14] AJCC Manual for Staging of Cancer 2002 6 175 Fifth Avenue, New York, NY, 10010, USA: Springer-Verlag New York, Inc

[B15] SEER-Medicare: defining the date of diagnosis & treatment[http://healthservices.cancer.gov/seermedicare/considerations/date.html]

[B16] Charlson ME Pompei P Ales KL MacKenzie CR A new method of classifying prognostic comorbidity in longitudinal studies: development and validation J Chronic Dis 1987 40 5 373 383 10.1016/0021-9681(87)90171-8 3558716

[B17] Klabunde CN Legler JM Warren JL Baldwin LM Schrag D A refined comorbidity measurement algorithm for claims-based studies of breast, prostate, colorectal, and lung cancer patients Ann Epidemiol 2007 17 8 584 590 10.1016/j.annepidem.2007.03.011 17531502

[B18] VassarStats: statistical computation web site[http://www.vassarstats.net/]

[B19] DePuy V Anstrom KJ Castel LD Schulman KA Weinfurt KP Saad F Effects of skeletal morbidities on longitudinal patient-reported outcomes and survival in patients with metastatic prostate cancer Support Care Cancer 2007 15 7 869 876 10.1007/s00520-006-0203-x 17262196

[B20] Norgaard M Jensen AO Jacobsen JB Cetin K Fryzek JP Sorensen HT Skeletal related events, bone metastasis and survival of prostate cancer: a population based cohort study in Denmark (1999 to 2007) J Urol 2010 184 1 162 167 10.1016/j.juro.2010.03.034 20483155

